# Longitudinal effect of CD4 by cotrimoxazole use on malaria incidence among HIV-infected Ugandan adults on antiretroviral therapy: a randomized controlled study

**DOI:** 10.1186/s12936-016-1426-z

**Published:** 2016-07-15

**Authors:** Ronnie Kasirye, Heiner Grosskurth, Paula Munderi, Jonathan Levin, Zacchaeus Anywaine, Andrew Nunn, Anatoli Kamali, Kathy Baisley

**Affiliations:** MRC/UVRI Uganda Research Unit on AIDS, Entebbe, Uganda; London School of Hygiene and Tropical Medicine, London, UK; School of Public Health, University of Witwatersrand, Johannesburg, South Africa; MRC Clinical Trials Unit at University College London, London, UK

**Keywords:** Malaria, CD4, Antiretroviral therapy, Cotrimoxazole, HIV

## Abstract

**Background:**

The effect of CD4 count on malaria incidence in HIV infected adults on antiretroviral therapy (ART) was assessed in the context of a randomized controlled trial on the effect of stopping cotrimoxazole (CTX).

**Methods:**

This study presents a sub-analysis of the COSTOP trial (ISRCTN44723643) which was carried out among HIV-infected Ugandan adults stable on ART with CD4 counts ≥250 cells/µl. Participants were randomized (1:1) to continue CTX or stop CTX and receive matching placebo, and were followed up for a minimum of 1 year (median 2.5 years). CD4 counts were measured at baseline, 3 months and then every 6 months. Clinical malaria was defined as fever and a positive blood slide. First, the relationship between current CD4 count during follow-up and malaria among participants on placebo was examined; using random effects Poisson regression to account for repeated episodes. Second, the effect of CD4 count at enrolment, CD4 count at ART initiation, and CD4 count during follow-up on malaria, was assessed within each trial arm; to examine whether the effect of CD4 count differed by CTX use.

**Results:**

2180 participants were enrolled into the COSTOP trial. The incidence of clinical malaria was approximately four episodes/100 person years in the CTX arm and 14 episodes/100 person years in the placebo arm. There was no evidence of an association of current CD4 and clinical malaria incidence (P = 0.56), or parasitaemia levels (P = 0.24), in the placebo arm. Malaria incidence did not differ by CD4 count at ART initiation, enrolment or during follow up, irrespective of CTX use. When compared with participants in the lowest CD4 stratum, rate ratios within each trial arm were all close to 1, and P values were all above P = 0.30.

**Conclusions:**

The immune status of HIV infected participants who are stable on ART as measured by CD4 count was not associated with malaria incidence and did not modify the effect of stopping CTX on malaria. The decision of whether to stop or continue CTX prophylaxis for malaria in HIV infected individuals who are stable on ART should not be based on CD4 counts alone.

*COSTOP trial registration number* ISRCTN44723643

**Electronic supplementary material:**

The online version of this article (doi:10.1186/s12936-016-1426-z) contains supplementary material, which is available to authorized users.

## Background

In many parts of sub-Saharan Africa, both malaria and HIV infection are highly endemic. HIV infection enhances malaria acquisition and severity; similarly malaria enhances HIV viral replication [1–5]. The effect of HIV infection on malaria incidence seems to be a consequence of the immune suppression that is a characteristic of HIV infection [6]. In clinical practice, CD4 cell counts are used to measure the degree of HIV-induced immune suppression which guides decisions on antiretroviral therapy (ART) and the need for prophylaxis against opportunistic infections [7, 8].

Decreased CD4 counts have been associated with higher risk of acquiring malaria. In a study in rural Uganda, HIV infected ART-naïve adults with CD4 counts <200 cells/µl had a significantly higher risk of clinical malaria than those with CD4 counts ≥500 [9]. A study in HIV infected ART-naïve adults in urban Entebbe, Uganda, found that the incidence of clinical malaria increased from 57/1000 person years (pyrs) among those with CD4 counts ≥500–140/1000 person years in those with CD4 <200 [10]. Both studies were done in the 1990s, before ART or cotrimoxazole (CTX) prophylaxis were routinely available in Uganda.

CTX is beneficial to HIV infected individuals as a prophylaxis against malaria and bacterial infections [11–13], even when individuals are on ART [14–16], and is recommended for routine use in areas where malaria is highly prevalent [8]. Once HIV viral replication is suppressed by ART, CD4 counts increase over time [17, 18]. Consequently it may be expected that the risk of malaria will decrease as individuals’ CD4 counts increase on ART. However, it is unclear whether the risk of malaria continues to decrease after the immune system has sufficiently recovered on ART, or whether there is a CD4 threshold after which malaria incidence stabilizes. It is also unclear whether decreased CD4 counts would be associated with an increased risk of malaria in individuals on CTX prophylaxis.

To address these research questions, we conducted a planned sub-group analysis to examine the relationship of CD4 count with malaria in the recently completed COSTOP trial in Uganda [19–21]. COSTOP was a randomized, placebo-controlled, non-inferiority trial assessing the efficacy and safety of stopping CTX prophylaxis among HIV positive adults who were stable on ART. The trial found that malaria incidence was higher among those who were randomized to stop prophylactic CTX [21]. Data from this trial were used to investigate the effect of CD4 count on the incidence of malaria, and whether the effect of CD4 count on malaria differed by CTX use.

### Aim

The study aimed to determine among HIV infected adults on ART with CD4 counts ≥250 cells/µl:

i. The effect of CD4 count on malaria incidence
ii. Whether this effect differs in the presence and absence of CTX medication

## Methods

This study used data gathered during the COSTOP trial conducted from 2011 to 2014 in Uganda (ISRCTN44723643). Trial methods have been described previously [19, 21]. Briefly, COSTOP was a randomized, double-blind, placebo controlled non-inferiority trial to determine whether long-term prophylaxis with CTX can be safely discontinued among HIV infected adults on ART with sustained immune competence (defined as a confirmed CD4 counts of ≥250 cells/µl). Individuals were eligible for enrolment if they were HIV-infected; aged 18 years or older; clinically asymptomatic; had been taking CTX and ART for at least 6 months; and had two CD4 counts (not more than 6 months apart) ≥250 cells/µl, the most recent no more than 4 weeks prior to enrolment. Exclusion criteria included pregnancy, grade 3 or 4 anaemia, neutropaenia or thrombocytopaenia. Participants were randomized to receive either active CTX (960 mg) or matching placebo once daily after stopping their regular CTX medication. Randomization was stratified by enrolment site (Entebbe or Masaka, both located in SW Uganda) and CD4 count (≥250–499 and ≥500 cells/µl).

### Study procedures

Informed consent for study procedures was obtained at screening and enrolment.

At screening, data were documented on; disease history, duration of prior ART and CTX medication, and CD4 count at time of ART initiation. At enrollment, data were documented on socio-demographic characteristics and each participant was provided with an insecticide-treated bed net (ITN) and educated about the importance of using it. Participants were seen at scheduled follow-up visits every month for the first 3 months and 3-monthly thereafter, and were followed for 12 months to 3.5 years, depending on date of enrolment. At these visits, participants were asked about their health, symptoms suggestive of malaria, adherence to medication and bed net use. Blood samples were drawn; at enrollment, monthly for 3 months and 3 monthly thereafter for a malaria slide; at 3 months, 6 months and 6 monthly thereafter for CD4 count; and 3 monthly for the full blood count. Participants were asked to attend the study clinic at any time they felt unwell; if malaria was suspected, based on a history of malaria associated symptoms (fever, headache, chills and rigors, joint aches, muscles aches, vomiting or diarrhea), a blood slide and other tests deemed necessary were done. Participants who reported having been treated for malaria elsewhere (for example during a journey) were asked to present documentary evidence of diagnoses and test results.

### Laboratory methods

A sample of blood was taken either from the fingertip using a lancet or from a peripheral vein using a syringe, and used to prepare thick and thin films on a glass slide. The specimens were processed using Leishman’s stain and examined by microscopy. Thick film specimens were used to record the number of parasites per 200 white blood cells and thin films to identify the plasmodium species. Venous blood samples were taken for CD4 cell counts and measured using a FACS-count system (Becton–Dickinson San Jose) at the MRC/UVRI laboratories in Entebbe and Masaka.

### Statistical analysis

Analyses were carried out using Stata 13. Clinical malaria was defined as presence or history (during the previous 2 weeks) of fever and microscopically confirmed malaria parasites. Severe malaria (based on WHO guidelines) [21, 22] was diagnosed if a participant had *Plasmodium falciparum* asexual parasitaemia, no other obvious cause of symptoms and met any of the following criteria: convulsions, loss of consciousness, hypotension (systolic blood pressure <70 mmHg), admission to hospital due to malaria, laboratory evidence of liver or kidney damage, severe normocytic anaemia (haemoglobin <50 g/dl, PCV < 15 %), or hyper parasitaemia on blood slide (>5 % or 250,000/µl).

The CD4 count at enrolment was calculated from the mean of the two most recent pre-enrolment (screening) CD4 counts. Person years at risk were calculated from enrolment until the date last seen or end of trial. After each malaria episode, participants were considered to be not at risk for another episode until the episode resolved, or for 28 days, if a resolution date was not available. Follow-up data were organized into intervals corresponding with the visit schedule. For time-varying variables during follow-up (e.g. CD4 count at malaria infection, BMI), the most recent value measured at the start of each interval was used. CD4 count values were carried forward for the visits where CD4 counts were not done, until the next recorded CD4 count.

First, the effect of current (time of infection) CD4 count on clinical malaria incidence during follow up was assessed; using random effects Poisson regression to account for the clustering of multiple episodes within the same participant. Since the incidence of malaria in the COSTOP trial had previously been shown to be significantly lower in the CTX arm (21), this analysis was restricted to participants in the placebo arm in order to examine the effect of CD4 counts in the absence of the anti-malarial effects of CTX. The effect of CD4 adjusted for baseline covariates that were considered as potential confounders a priori (enrolment site, age, sex, socioeconomic status (SES) and baseline CD4 count) was examined, and then including time-varying variables (time since enrolment, current BMI). SES was measured by combining baseline data from all trial participants on housing construction and ownership of household items into an asset index score using principal component analysis [23]. In order to allow for non-linear effects, CD4 at infection, baseline CD4 and age were modelled using restricted cubic splines with 4 knots; this approach provides a flexible way to model the shape of the relationship of a continuous variable with the outcome [24].

Among placebo participants with clinical malaria, the effect of CD4 count at infection on parasitaemia during each malaria episode as the outcome was assessed, using random effects linear regression; parasitaemia levels were log transformed for analysis. The analysis was adjusted for baseline and time-varying potential confounders as described above. In addition, CD4 count at infection was assessed for an effect on severe malaria; since there were only 15 episodes of severe malaria (13 placebo, two on CTX) [21], no attempt was made to adjust for potential confounders.

Secondly, the effect of CD4 count at baseline, CD4 count at ART initiation, or CD4 count at infection on clinical malaria was assessed by treatment arm (CTX or placebo), using random effects Poisson regression. Regression models contained fixed effects for CD4 count group, treatment arm, enrolment site and year since enrolment, and an interaction term between CD4 count group and treatment arm.

## Results

2180 participants were enrolled into the COSTOP trial, 1002 (46 %) at the Entebbe site, and 1091 (50 %) were allocated to placebo (stopping CTX). Baseline characteristics were well balanced between trial arms (Additional file 1). Mean age at enrolment was 41 years and 74 % were female. The median (IQR) CD4 count at ART initiation was 155 (89–199) and 159 (83–214) for Entebbe and Masaka, respectively, and the median (IQR) CD4 count at enrolment was 446 (361–600) and 519 (397–655), respectively. At the Entebbe site, 56 (5.7 %) participants were on a protease inhibitor (PI)-containing regimen compared to 30 (2.6 %) at Masaka. 239 (24 %) participants in Entebbe and 220 (19 %) in Masaka site had been on ART for <2 years.

### Effect of CD4 on malaria

**Fig. 1:**
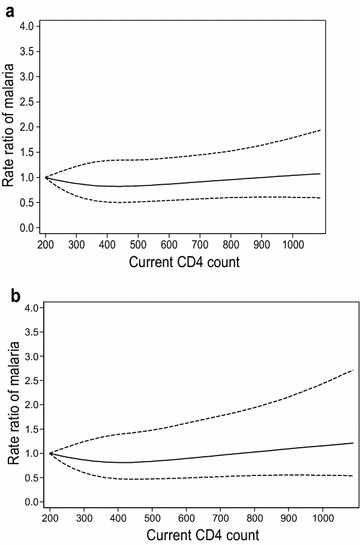
Association of malaria incidence rate ratios (and 95 % confidence intervals) with CD4 count at time of malaria episode as observed during follow up, modelled using restricted cubic splines with 4 knots in a random effects Poisson regression model, unadjusted (**a**), and adjusted for covariates during baseline and follow up (**b**). A CD4 count of 200 was used as the reference to calculate the rate ratios

**Fig. 2:**
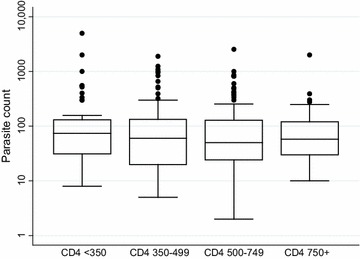
Parasite counts by CD4 count at infection among participants in the placebo arm with clinical malaria. The *central line* represents the median; *boxes* represent 75th and 25th centiles; *whiskers* represent* upper* and* lower* adjacent values and *dots* represent outside values

Among participants in the placebo arm, overall clinical malaria incidence was 14.1/100 person years (95 % CI 12.5–15.8). There was no evidence of an effect of CD4 count at infection on clinical malaria (P = 0.56; Table 1; Fig. 1). Furthermore, there was no evidence that parasitaemia levels differed by CD4 count at infection (P = 0.24 from random effects linear regression model; Fig. 2).

**Table 1:** Association of CD4 count (at infection) with malaria in the placebo arm

	Clinical malaria				
	Median value	Rate/100 person years (95 % CI)^a^	Crude rate ratio (95 % CI)^a^	Adjusted rate ratio (95 % CI)^a,b^	Adjusted rate ratio (95 % CI)^a,c^
CD4 count at infection^d^			P = 0.60	P = 0.81	P = 0.56
<300	262	14.6 (11.2–19.0)	1	1	1
300–399	355	13.4 (11.0–16.3)	0.9 (0.7–1.1)	0.9 (0.7–1.2)	0.9 (0.7–1.2)
400–499	448	13.1 (11.1–15.5)	0.9 (0.7–1.2)	0.9 (0.7–1.2)	0.9 (0.6–1.3)
500–599	547	13.5 (11.5–15.9)	0.9 (0.7–1.3)	0.9 (0.6–1.3)	0.9 (0.6–1.4)
600–699	644	14.2 (11.7–17.1)	1.0 (0.7–1.4)	0.9 (0.6–1.5)	1.0 (0.6–1.6)
≥700	836	15.5 (12.7–19.0)	1.1 (0.8–1.5)	1.0 (0.6–1.7)	1.2 (0.7–1.9)
Baseline factors					
Site			P < 0.001	P < 0.001	P = 0.002
Entebbe		10.6 (8.8–12.8)	1	1	1
Masaka		17.2 (14.9–19.9)	1.6 (1.3–2.1)	1.5 (1.2–1.9)	1.5 (1.1–1.9)
Age (years)^d^			P = 0.07	P = 0.04	P = 0.04
<35	31	12.4 (10.0–15.2)	1	1	1
35–44	39	15.1 (12.8–17.9)	1.2 (1.0–1.5)	1.2 (1.0–1.5)	1.2 (1.0–1.5)
≥45	50	14.0 (11.5–17.1)	1.1 (0.8–1.5)	1.1 (0.8–1.4)	1.1 (0.8–1.5)
Sex			P = 0.83	P = 0.46	P = 0.63
Male		14.4 (11.4–18.1)	1	1	1
Female		13.9 (12.2–16.0)	1.0 (0.7–1.3)	0.9 (0.7–1.2)	0.9 (0.7–1.2)
SES			P < 0.001	P = 0.002	P = 0.003
Low		16.1 (13.6–19.0)	1.8 (1.3–2.4)	1.7 (1.3–2.4)	1.7 (1.2–2.3)
Middle		15.7 (12.8–19.1)	1.7 (1.2–2.4)	1.6 (1.1–2.2)	1.6 (1.1–2.2)
High		9.0 (6.9–11.8)	1	1	1
Baseline CD4 count^d^			P = 0.63	P = 0.88	P = 0.98
<350	310	13.3 (10.4–16.9)	1	1	1
350–499	422	13.1 (10.7–16.1)	1.0 (0.7–1.3)	1.0 (0.7–1.3)	1.0 (0.7–1.4)
≥500	634	14.7 (12.1–18.0)	1.1 (0.8–1.5)	1.0 (0.8–1.4)	1.0 (0.6–1.5)
Factors during follow-up					
Time since enrolment (years)			P < 0.001		P < 0.001
<1		17.3 (14.8–20.1)	1.9 (1.4–2.6)		1.9 (1.4–2.7)
1–2		13.1 (10.8–15.7)	1.4 (1.0–2.0)		1.5 (1.0–2.1)
≥2		9.0 (6.7–12.1)	1		1
BMI (kg/m^2^)			P = 0.01		P = 0.02
<18.5		10.0 (6.9–14.4)	0.6 (0.4–0.9)		0.6 (0.4–0.9)
18–24.9		15.8 (13.8–18.1)	1		1
≥25		11.6 (8.9–15.0)	0.7 (0.5–1.0)		0.8 (0.6–1.0)
Bed net use			P = 0.05		P = 0.14
≥90 % of visits		13.2 (11.6–15.1)	1		1
<90 % of visits		17.5 (13.7–22.3)	1.3 (1.0–1.7)		1.2 (0.9–1.6)

^a^Rates and rate ratios estimated from random effects Poisson regression

^b^Adjusted for enrolment site, age at enrolment, sex, SES and baseline CD4 count

^c^Adjusted for all covariates in footnote b, and time since enrolment, current BMI and bednet use

^d^Continuous covariates (CD4 count and age) were modelled by restricted cubic splines with 4 knots. Rates are estimated at the median value in each range; the median value in the lowest range is used as the reference to estimate the rate ratios. P value is for overall association with covariate from likelihood ratio test

There were 15 cases of severe malaria (13 placebo, 2 CTX). Severe malaria rates decreased with increasing CD4 counts among participants with CD4 <400, then remained fairly similar in participants with higher CD4 counts. (P = 0.14; Additional file 2).

### Effect of CD4 count on malaria by trial arm

The incidence of malaria did not differ significantly between CD4 count strata, neither for CD4 count at infection, CD4 count at ART initiation or CD4 count at enrolment into the study, and this was irrespective of whether participants were in the CTX arm or the placebo arm of the trial. Although malaria incidence was significantly lower in the CTX arm than on placebo [21], compared to participants on the lowest CD4 stratum, rate ratios were all close to 1 and P values were all above P = 0.30 for each of the three CD4 measures and within each arm (Table 2).

**Table 2:** Effect of trial drug on malaria, by CD4 count at enrolment, ART initiation and at the time of malaria episode

Trial arm	Stratum	Episodes	Person years	Rate/100 person years (95 % CI)^a^	Rate ratio^b^	P value^c^
CD4 count at ART initiation						
CTX	<100	30	698	4.0 (2.8–5.9)	1	0.99
	100–249	59	1461	3.9 (3.0–5.1)	1.0 (0.6–1.5)	
	250+	10	229	3.9 (2.0–7.4)	1.0 (0.5–2.0)	
Placebo	<100	92	709	12.4 (9.8–15.7)	1	0.73
	100–249	196	1458	13.4 (11.4–15.7)	1.1 (0.8–1.4)	
	250+	36	212	14.7 (10.1–21.5)	1.2 (0.8–1.9)	
CD4 count at enrolment						
CTX	<350	14	416	3.5 (2.0–5.9)	1	0.51
	350–499	32	900	3.4 (2.4–4.8)	1.0 (0.5–1.9)	
	500+	57	1234	4.3 (3.3–5.7)	1.3 (0.7–2.3)	
Placebo	<350	58	453	13.1 (9.8–17.5)	1	0.98
	350–499	117	850	13.6 (11.1–16.7)	1.0 (0.7–1.5)	
	500+	175	1211	13.4 (11.3–16.0)	1.0 (0.7–1.4)	
CD4 count at infection						
CTX	<350	21	4618	4.4 (2.9–6.9)	1	0.52
	350–499	30	8811	3.3 (2.3–4.7)	0.7 (0.4–1.3)	
	500+	52	1197	4.0 (3.0–5.4)	0.9 (0.5–1.5)	
Placebo	<350	60	390	14.6 (11.1–19.2)	1	0.32
	350–499	98	807	11.8 (9.5–14.6)	0.8 (0.6–1.1)	
	500+	192	1317	14.1 (12.0–16.5)	1.0 (0.7–1.3)	

^a^Marginal means from random effects Poisson regression model with fixed effects for CD4 count stratum, treatment arm and their interaction, and site and year since enrolment

^b^Rate ratio for effect of treatment arm in each CD4 count stratum, adjusted for site and year since enrolment, from random effects Poisson regression model

^c^P values for overall association of CD4 count with malaria incidence within each treatment arm. P values for interaction between CD4 count and treatment arm: CD4 count at ART initiation P = 0.87; CD4 count at enrolment P = 0.60; CD4 count at infection P = 0.96

## Discussion

Previous studies in HIV-infected adults have reported an increase in malaria incidence with decreasing CD4 counts [9, 10], but these studies were in individuals who were not on ART.

The incidence of clinical malaria in COSTOP trial participants was lower in the CTX arm compared to placebo, and reduced during follow up [21]. This reduction over time was primarily driven by reduced incidence in the placebo arm while incidence in the CTX arm remained fairly constant. One possible explanation is that the immune system recovers in individuals on ART and is, therefore, able to more effectively control malaria infection. In the COSTOP trial, there was evidence of continued recovery of the immune system in HIV-infected participants who are stable on ART as shown by an increase in CD4 counts over time, particularly in participants on placebo [20]. However, no evidence was found of the expected association between CD4 count and the incidence of clinical malaria, or degree of parasitaemia. This lack of an effect of CD4 count on malaria was observed for CD4 count at the time of starting ART (considered a measure of the extent of immune damage before starting ART), time of randomization (indicating the immune status at beginning of study) and time of malaria episode, in participants who continued CTX prophylaxis and in those who stopped. Results from this study are consistent with those of a recent unblinded trial of CTX discontinuation in adults on ART in Kenya, which found that the effect of stopping CTX on malaria was similar in participants with CD4 count ≤600 at enrolment and those with CD4 >600 [25]. However, there were very few malaria episodes in that trial (34 in total), and the authors did not directly examine the relationship between CD4 count and malaria. One possible explanation for these findings is that there is a threshold below which CD4 count significantly influences the risk of malaria. All participants in the COSTOP trial had a CD4 count ≥250 cells/µl at enrolment, and those in the Kenya trial had a CD4 count >350. An alternative explanation could be that an improvement of CD4 cell quality rather than quantity under ART may be important for malaria containment [26]. In this study, there was no evidence of an effect of CD4 count on severe malaria; however, because there were so few cases of severe malaria, our power to detect significant associations was poor.

### Strengths and limitations of the study

This study made use of a well-documented data set from a large trial of HIV-infected adults on ART. The large sample size and the regular collection of data on exposures (CD4 count), outcomes (clinical malaria and parasitaemia) and a variety of potential confounders made it possible to investigate the research questions in great detail.

This study had some limitations. In spite of the large sample size only a small number of severe malaria episodes occurred which limited the power to detect a potential effect of CD4 count. Also, the study was not a priori designed to address detailed research questions related to malaria, but rather was conducted as a sub-analysis of data gathered in the context of a randomized trial on the effect of stopping CTX. Although some potential confounders were adjusted for, residual confounding cannot be ruled out as a result of imperfectly measured covariates which were adjusted for (e.g. SES) or covariates which were not measured. Furthermore, viral load, which may be a better indicator of immune competence than CD4 count was not measured: it has been shown that effective viral suppression reduces the incidence of opportunistic infections [27] and a similar effect might be expected for clinical malaria. Lastly, it was not possible to investigate the immune responses to malaria which might have provided insight into why CD4 counts had no apparent effect on malaria incidence.

## Conclusions

In this study of HIV-infected individuals on ART with baseline CD4 counts ≥250 cells/µl, the incidence of clinical malaria and the intensity of parasitaemia among patients with clinical malaria were not influenced by CD4 counts at ART initiation, enrollment into the study, or at the time of malaria infection. The finding of no association between malaria and CD4 count was similar among participants randomized to stop prophylactic CTX and those who continued CTX. The decision of whether to stop or continue CTX prophylaxis for malaria in HIV infected patients who are stable on ART should not be based on a patient’s CD4 cell count alone.

## Additional files

10.1186/s12936-016-1426-z Rate ratio and 95 % confidence interval for change in incidence of severe malaria with CD4 count at infection during follow-up modelled using restricted cubic splines with 3 knots in a Poisson regression model (Note: no participant had more than one event so random effects not included).
10.1186/s12936-016-1426-z Baseline characteristics by trial arm and site.
