# Supplementary material for: Longitudinal effect of CD4 by cotrimoxazole use on malaria incidence among HIV-infected Ugandan adults on antiretroviral therapy: a randomized controlled study
Source: Malar J. 2016 Jul 15;15:361. doi: 10.1186/s12936-016-1426-z (PMC4946223; doi:10.1186/s12936-016-1426-z)
Supplement: Supplementary file 1 — 10.1186/s12936-016-1426-z Rate ratio and 95 % confidence interval for change in incidence of severe malaria with CD4 count at infection during follow-up modelled using restricted cubic splines with 3 knots in a Poisson regression model (Note: no participant had more than one event so random effects not included). [file 12936_2016_1426_MOESM1_ESM.docx]

Table 1

|  | **Overall (N=2180)** | | **Site** | | | | | |
| --- | --- | --- | --- | --- | --- | --- | --- | --- |
|  |  |  | **Entebbe** | | | **Masaka** | | |
|  | **CTX (N=1089)** | **Placebo (N=1091)** | **CTX**  **(N=501)** | **Placebo**  **(N=501)** | **Total  (N=1002)** | **CTX**  **(N=588)** | **Placebo**  **(N=590)** | **Total  (N=1178)** |
| **Age** |  |  |  |  |  |  |  |  |
| Mean (SD) | 41.0 (8.0) | 40.7 (8.3) | 40.2 (7.9) | 39.9 (8.1) | 40.1 (8.0) | 41.7 (8.1) | 41.3 (8.5) | 41.5 (8.3) |
| **Sex** |  |  |  |  |  |  |  |  |
| Male | 286 (26.3) | 283 (25.9) | 130 (26.0) | 125 (25.0) | 255 (25.5) | 156 (26.5) | 158 (26.8) | 314 (26.7) |
| Female | 803 (73.7) | 808 (74.1) | 371 (74.0) | 376 (75.0) | 747 (74.6) | 432 (73.5) | 432 (73.2) | 864 (73.3) |
| **Socio-economic status** | |  |  |  |  |  |  |  |
| Low | 486 (44.6) | 474 (43.4) | 197(39.3) | 213 (42.5) | 410(40.9) | 289 (49.1) | 261 (44.2) | 550 (46.7) |
| Medium | 318 (29.2) | 329 (30.2) | 117 (23.4) | 113 (22.6) | 230(23.0) | 201 (34.2) | 216 (36.6) | 417 (35.4) |
| High | 285 (26.2) | 288 (26.4) | 187 (37.3) | 175 (33.9) | 362(36.1) | 98 (16.7) | 113 (19.2) | 211 (17.9) |
| **CD4 count at enrolment** | |  |  |  |  |  |  |  |
| <350 | 181 (16.6) | 201 (18.4) | 110 (22.0) | 110 (22.0) | 220 (22.0) | 71 (12.1) | 91 (15.4) | 162 (13.8) |
| 350-499 | 394 (36.2) | 371 (34.0) | 182 (36.3) | 189 (37.7) | 371 (37.0) | 212 (36.1) | 182 (30.8) | 394 (33.4) |
| ≥ 500 | 514 (47.2) | 519 (47.6) | 209 (41.7) | 202 (40.3) | 411 (41.0) | 305 (51.9) | 317 (53.7) | 622 (52.8) |
| **ART regimen at enrolment**^1^ | |  |  |  |  |  |  |  |
| NNRTI | 1021 (95.1) | 1025 (94.9) | 4532 (92.6) | 449 (91.5) | 902 (92.0) | 568 (97.1) | 576 (97.8) | 1144 (97.4) |
| NRTI | 11 (1.0) | 11 (1.0) | 11 (2.3) | 11 (2.2) | 22 (2.2) | 0 | 0 | 0 |
| PI | 42 (3.9) | 44 (4.1) | 25 (5.1) | 31 (6.3) | 56 (5.7) | 17 (2.9) | 13 (2.2) | 30 (2.6) |
| **Years on ART**^1^ |  |  |  |  |  |  |  |  |
| < 1 | 73 (6.7) | 82 (7.5) | 46 (9.2) | 43 (8.6) | 89 (8.9) | 27 (4.6) | 39 (6.6) | 66 (5.6) |
| 1 to 2 | 144 (13.2) | 160 (14.7) | 71 (14.2) | 79 (15.8) | 150 (15.0) | 73 (12.4) | 81 (13.7) | 154 (13.1) |
| 2 to 5 | 508 (46.7) | 480 (44.0) | 261 (52.0) | 255 (50.9) | 516 (51.5) | 247 (42.0) | 225 (38.2) | 472 (40.1) |
| >5 | 364 (33.4) | 369 (33.8) | 123 (24.6) | 124 (24.7) | 247 (24.6) | 241 (41.0) | 245 (41.5) | 486 (41.3) |
| **CD4 count at ART initiation**^2^ | |  |  |  |  |  |  |  |
| <100 | 292 (28.7) | 310 (30.2) | 135 (28.8) | 139 (28.5) | 274 (28.6) | 157 (28.7) | 171 (31.7) | 328 (30.1) |
| 100-249 | 619 (60.9) | 621 (60.4) | 296 (63.1) | 314 (64.3) | 610 (63.7) | 323 (58.9) | 307 (56.8) | 630 (57.9) |
| ≥ 250 | 106 (10.4) | 97 (9.4) | 38 (8.1) | 35 (7.2) | 73 (7.6) | 68 (12.4) | 62 (11.5) | 130 (12.0) |

^1^Missing data on ART regimen at enrolment for 26 participants (15 on CTX and 11 on placebo). ^2^Missing data on CD4 count at ART initiation for 135 participants, 72 on CTX and 63 on placebo.
