# Supplementary material for: Longitudinal effect of CD4 by cotrimoxazole use on malaria incidence among HIV-infected Ugandan adults on antiretroviral therapy: a randomized controlled study
Source: Malar J. 2016 Jul 15;15:361. doi: 10.1186/s12936-016-1426-z (PMC4946223; doi:10.1186/s12936-016-1426-z)
Supplement: Supplementary file 2 — 10.1186/s12936-016-1426-z Baseline characteristics by trial arm and site. [file 12936_2016_1426_MOESM2_ESM.docx]

**Figure 1:**
